# Detection of autism spectrum disorder (ASD) in children and adults using machine learning

**DOI:** 10.1038/s41598-023-35910-1

**Published:** 2023-06-13

**Authors:** Muhammad Shoaib Farooq, Rabia Tehseen, Maidah Sabir, Zabihullah Atal

**Affiliations:** 1 Department of Artificial Intelligence, University of Management and Technology, Lahore, 54000 Pakistan; 2 Department of Computer Science, University of Central Punjab, Lahore, 54000 Pakistan; 3 Department of Computer Science, Kardan University, Kabul, 1007 Afghanistan

**Keywords:** Neurological disorders, Diseases, Neurology

## Abstract

Autism spectrum disorder (ASD) presents a neurological and developmental disorder that has an impact on the social and cognitive skills of children causing repetitive behaviours, restricted interests, communication problems and difficulty in social interaction. Early diagnosis of ASD can prevent from its severity and prolonged effects. Federated learning (FL) is one of the most recent techniques that can be applied for accurate ASD diagnoses in early stages or prevention of its long-term effects. In this article, FL technique has been uniquely applied for autism detection by training two different ML classifiers including logistic regression and support vector machine locally for classification of ASD factors and detection of ASD in children and adults. Due to FL, results obtained from these classifiers have been transmitted to central server where meta classifier is trained to determine which approach is most accurate in the detection of ASD in children and adults. Four different ASD patient datasets, each containing more than 600 records of effected children and adults have been obtained from different repository for features extraction. The proposed model predicted ASD with 98% accuracy (in children) and 81% accuracy (in adults).

## Introduction

Autism is categorized as neuro-developmental disorder which has severe effects on social growth and development in children and adults. Although its complete cure seems not possible but early diagnosis is preferable as it helps in more effective treatment compared to conventional behavioural investigations that take much time in detecting and diagnosing ASD by analysing children behaviour in clinics^1^. ASD has been mostly diagnosed in 2 years old child but it can be diagnosed in children later depending on complexity of symptoms and severity of the disorder^2^. It has generally occurred due to environmental factors or any genetic linkage which not only effects the nervous system but also has an overall impact on social and cognitive skills of the children and adults. The extent and the intensity of its symptoms are quite variable. Common signs of the condition include difficulty in communication particularly in social situations, obsessional interests, and repeated mannerisms^3^. A complete examination is needed to detect ASD comprising thorough evaluation and series of assessments performed by child healthcare professionals and psychologists. Early treatment and diagnosis of ASD are crucial since they help to somewhat lessen symptoms, which enhances the person's overall quality of life^4^. However, a lot of critical time can be lost in diagnosing ASD because it cannot be properly detected by depicting only behaviours of children or adults in clinic. Autism can be identified as early as possible using a range of clinical approaches, but actually these are time-consuming diagnostic procedures infrequently carried out unless the predictive risk of ASD development is high^5^. Machine learning (ML) gives an opportunity to train ASD models in less time and more accuracy^6^. ML techniques are crucial for quick and accurate assessment of ASD risk and streamlining the entire diagnostic process which assist families in getting to the critical therapies more quickly^7^. Various classification models of ML can be used for early prediction of autism to prevent its prolonged effects in adults as well as children^8^.

Many other computational techniques have also been proposed in literature^9^ such as Hosseinzadeh et al.^10^ proposed IoT based solution for ASD detection and Eslami and Saeed^11^ presented deep learning based model for healthcare of ASD effected patents. However, obtaining huge amount of data for model training in centralized or distributed environment remained a challenge. Hospitals hesitate to share their data as data are the most valuable asset and regional data protection legislations also prohibit data sharing^12^. Data owner organizations have many serious concerns about data privacy, data security and data protection. Moreover, transmission of big dataset over the network for training machine learning model introduces further barriers of network latency, communication delay and data theft^13^. Therefore, it is the immense need of time that a model should be proposed in which data remain safe with owner organization.

Federated Learning (FL) technique is the most advanced approach of ML in which data remains secure with owner organization and small sized local ML based classifier is trained onsite without moving data over the network^14^. FL is very beneficent in ensuring data security as data are not being shared over the network therefore data privacy, data protection and data security issues are automatically resolved^15^. Moreover, network issues will not be raised as only small sized local data model is travelling over the network towards central server instead of huge data^16^. Many researchers have applied FL for detection of multiple neurological disorders^17^. Ali et al.^18^ have applied FL for the detection of colon cancer using pixel level segmentation dataset. Ghosh et al.^19^ have applied FL for medical image segmentation. Nigmatullina et al.^17^ proposed a digital platform to monitor and support children with ASD using FL. Novelty of our work is the application of FL technique for detection of ASD in both children and adults. Two different ML models including SVM and LR have been trained locally using four different ASD datasets of features containing records about children and adults obtained from free sources and data providing agencies listed in Table 1 for autism detection. We have also compared the results of proposed model with already proposed ASD detection methods and comparable accuracy has been obtained. Major contribution of this work is the combination of different local ML based models for training central FL based meta classifier on features dataset of children and adults to detect ASD risk factors with reasonable accuracy.

**Table 1:** Dataset description of children and adults.

Categories	Source	No. of instances	No. of attributes
Children	A: https://archive.ics.uci.edu/ml/datasets/Autistic+Spectrum+Disorder + Screening + Data + for + Children++	692	21
	B: https://www.kaggle.com/datasets/fabdelja/autism-screening-for-toddlers	654	19
Adults	C: https://archive.ics.uci.edu/ml/datasets/Autism+Screening+Adult	704	21
	D: https://www.kaggle.com/datasets/andrewmvd/autism-screening-on-adults	700	19

Our article is organized in multiple sections. In “Introduction” section presents introduction of the autism detection approaches. Most recent studies conducted on autism detection have been summarized in “Related work” section. Research methodology, experimentation, analysis and results have been presented in “Material and method” section. Results have been discussed in “Discussion” section. Conclusion and future directions have been illustrated in “Conclusion” section.

## Related work

Autism spectrum disorder (ASD) is a neuro-developmental disorder that results various impairments in social interaction, communication, and the existence of unvaried patterns of behaviour in children and adults^20^. Alfalasi^21^ reported that in United States 1 out of 54 children is affected by autism. Detecting autism earlier in one life can make a big difference than treating it later^22^. According to World Health Organization (WHO) every year one among 160 children is diagnosed with ASD traits all over the world^23^. Treating ASD earlier is always the best option for toddlers as they are still developing^24^.

Different symptoms identified in ASD patients have been considered as features that can be used for ASD detection. Lawan et al.^25^ and Cantin-Garside et al.^26^ observed behavioural disorder, Beary et al.^27^ and Derbali et al.^28^ recorded facial expression disorder and Devika et al.^29^ observed structural disorder in ASD effected persons. Emotional disorder in ASD affected persons has been studied by Makhnytkina et al.^30^ and mental disorder has been analysed in Liu et al.^31^ and Lord et al.^32^. Many researchers explored medical imageries for ASD detection including Bilic et al.^33^, Husna et al.^34^, Liu et al.^35^, Nogay and Adeli^36^. Images of brain have been used by Subah et al.^37^, Xu et al.^38^, Yin et al.^39^, Shenouda et al.^40^ to detect ASD in patients. Single and cross order strategy for ASD detection has been proposed in Wawer et al.^41^.

Researchers have used wearable devices containing sensors for detection of ASD^42,43^. Application of intelligent approaches present advanced ways to economically detect ASD effected children and adults^44^. Models have been proposed in the literature describing application of different methods and approaches for ASD detection like structural MRI^45^, neural networks^46^, machine learning^47–49^, deep learning^50^, transfer learning^51,52^ and IoT ^53^. All these techniques have been applied to detect ASD with reasonable accuracy in children and adults but faced limitations of data acquisition as hospitals hesitate or refuse to share patient records due to organizational policies and regional data protection legislations. Data security, data privacy and data availability are the huge challenges in developing effective intelligent models. Even if access to data is granted, transferring huge dataset over the network is again challenging, rising a lot of network issues regarding network congestion, latency and data theft.

Federated learning (FL) provides a generous solution to address all above mentioned problems. FL is an advanced ML based approach that never transmits data over the network^54^. Data is kept with its generating organization^55^ whereas only a small sized local data model is trained from onsite data and transmitted over the network towards central server where all local models are combined to train meta classifier for determining which ML model is most effective in autism detection^56^. Objective of proposed model is to detect ASD symptoms at different stages of age with minimum time, controlled expense and maximum accuracy. Novelty of our work is the application of federated learning technique for autism detection in children and adults by processing four different datasets by training SVM and LR classifiers locally. Major contribution of this work is the detection of ASD by the application of most advanced Federated Learning technique by training ML classifiers locally on features dataset of children and adults to find the predictive risk factors of Autism with reasonable accuracy.

## Material and method

**Figure 1:**
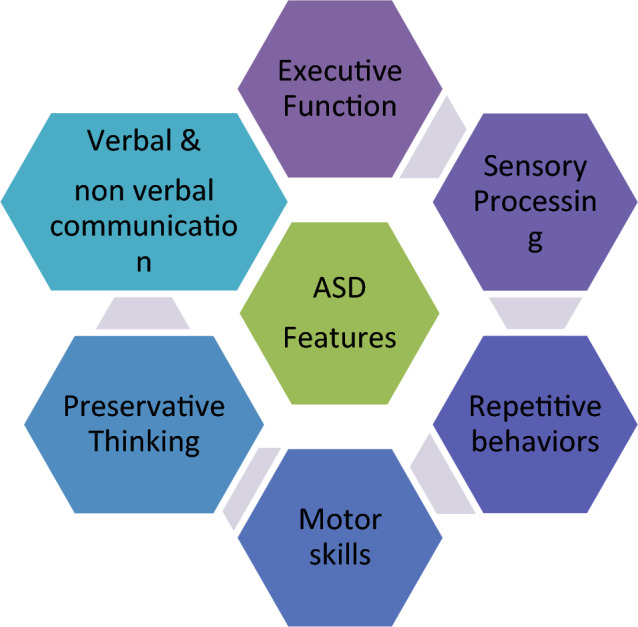
Aspects observed while diagnosing ASD.

ASD indicates a disability in human development due to variations of neurons present in human brain^57^. Practitioners believe that there are multivariate sources that work jointly to cause ASD^58^. Diagnosis of ASD is also very challenging task as no medical test like blood test exists to detect ASD. Doctors usually apply psychological and observational strategies to sense ASD in a patient by analysing multiple aspects of their daily routine as mentioned in Fig. 1.

**Figure 2:**
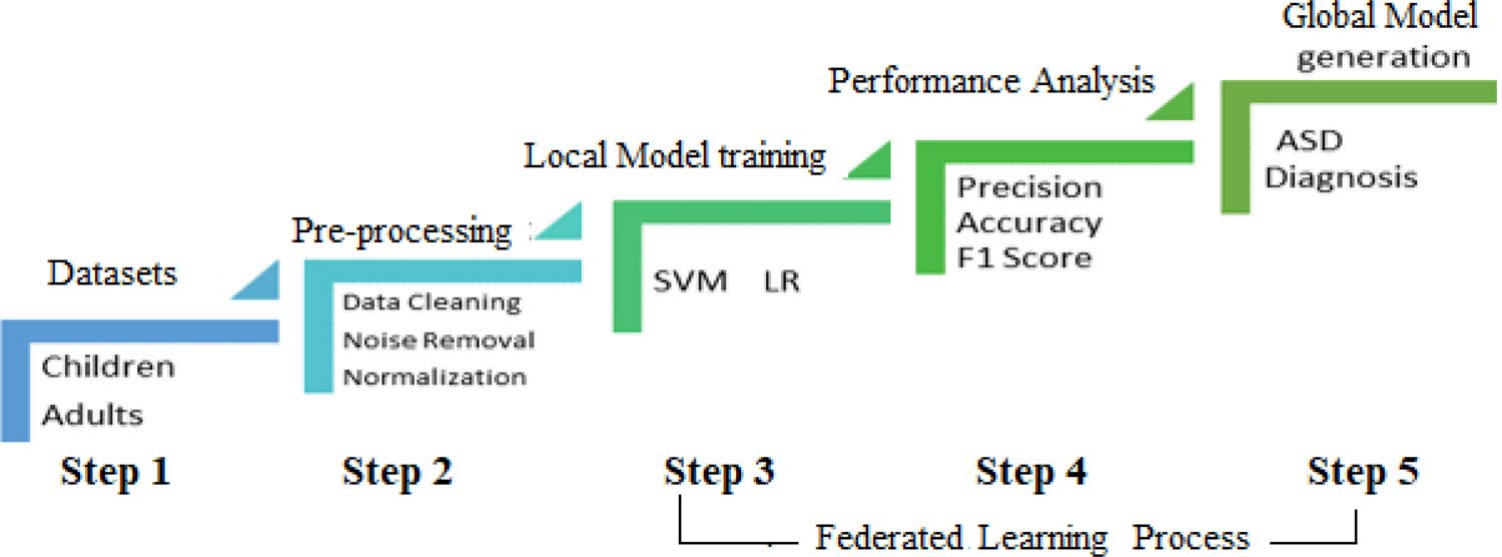
Proposed model architecture.

In this article, a unique federated learning based model has been proposed in which four different datasets of adults and children have been analysed using LR and SVM locally to train local data models. These local models have been transmitted towards central server for training of meta classifier in global model to predict autism in children and adults. Proposed model architecture presented in Fig. 2 comprises of five components including dataset acquisition, data pre-processing, ML models training for ASD detection and performance comparison of different ML models to determine the most effective model that can accurately diagnose autism. The first step was acquisition of data in which publicly available four datasets of children and adults from data sources listed in Table 1 have been obtained. In second step, data pre-processing and normalization was performed for data compression and data cleaning and removal of noisy data. After normalization, in third step, four datasets have been locally processed by SVM and LR classifiers for autism detection. Results of training ML classifiers have been transmitted to central server where meta classifier has been trained to compare results and identify the best model to detect autism. In last step, results of meta classifier were validated by calculating accuracy, precision and F1 score to detect autism disorder with more accuracy as shown in Fig. 2.

*Step1: Dataset*

Four datasets have been obtained covering two dimensions: children and adults. Source and specifications of each dataset is listed in Table 1.

*Step 2: Pre-processing*

According to Q-Chart-10, ten different features have been unanimously identified for processing of adults and children datasets at same scale for segregation of autism effected patients from normal ones as shown in Table 2.

**Table 2:** Feature description.

Feature no	Title	Description	Response
1	Patient’s age	Children (0–15 years), adults (16 years or above)	R1
	Patient’s gender	Male/female	
	Ethnicity of patient	Common ethnicities	
	Residence country	Countries list	
2	Verbal communication	Response on calling Name, saying papa, mama	R2
3	Non-verbal communication	Eye contact, facial expressions, gestures, posture, use of objects and body language	R3
4	Sensory processing	See, hear, smell, taste, touch	R4
5	Repetitive behaviour	Do action again and again	R5
6	Motor skills	Walking, running, riding a bike	R6
7	Preservative thinking	Rumination, repetitive thinking, worry	R7
8	Having jaundice	Child/adult born with jaundice	R8
9	Person taking ASD test	Parent staff, caregiver etc.	R9
10	Used the screening app before	Did the user use a screening app for ASD	R10
	Screening method type	Type of methods of screening chosen based on age category	

The Quantitative Checklist for Autism in Children (Q-CHART-10) screening approach approved by Transforming autism project, UK, served as the foundation for the conduction of this research^3^ . Thirty questions have been asked to record responses (R1–R10) for features mentioned in Table 2. The value of these responses is assigned to classes as per following criteria for assigning weightage (score) to every response.
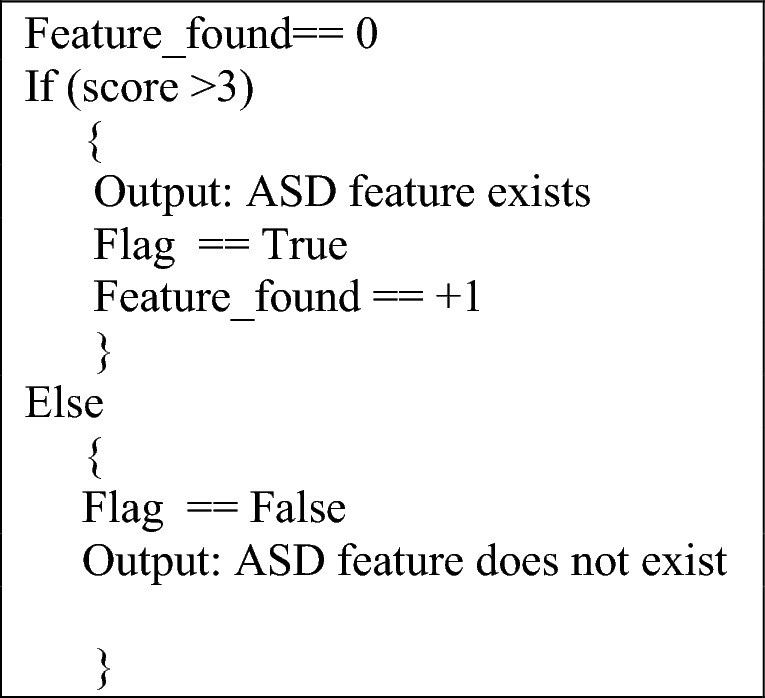


If score of class is more than 3, it indicates that ASD feature exits, its weight is incremented by 1 and “Yes” will be stored in response set otherwise value of flag will remain 0 that shows absence of any ASD features and “No” will be stored in the response set. Each class variable corresponds to more than one questions confirming the presence of feature extracted from Q-CHART-10 checklist. Information stored in class response set is in the binary format indicating Yes (stored as 1) and No (stored as 0). Local ML models have been trained on these responses presented in Table 2.

The response dataset contained some noisy and missing records therefore data transformations were needed to carry out prior to train ML classifier for model training and analysis. Category variables are handled using label encoding. To make labels machine-readable, label encoding transforms them into numeric form. Repeated labels receive the same value as those that were previously allocated. The binary label encoding of classes with ten features have been chosen.

*Step 3: Federated Learning process*

**Figure 3:**
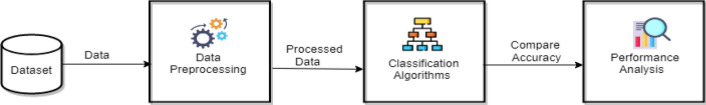
Proposed model workflow.

In the proposed architecture, Federated learning process starts from step three in which pre-processed and normalized datasets have been processed for training of SVM and LR classifiers. Workflow of FL process is presented in Fig. 3. Results of these classifiers in terms of accuracy, precision and F1 score have been calculated and transmitted to central server for training of meta classifier at server. Meta classifier will determine which model is more appropriate in detecting autism and will train the global model accordingly. Global model will be disseminated in all clients as a single tool for autism detection.

### Experiment

The children and adult datasets (A, C respectively) presented in Table 1 have been divided into training and test datasets. Training datasets contained 80% records and testing datasets which will be used to test the proposed model contained 20% of total records.

#### Experimental setup

**Figure 4:**
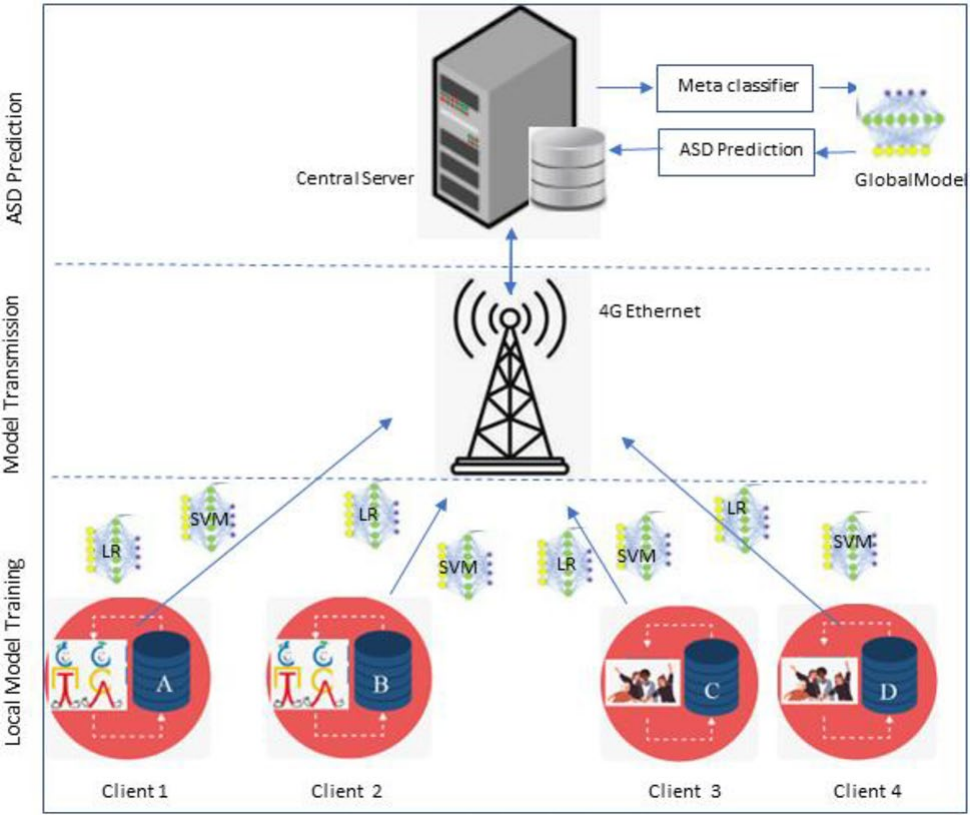
Experimental setup.

Experiment has been performed in two different dimensions. In first dimension, SVM and LR has been applied on dataset of adults presented in Table 1. In second dimension, SVM and LR has been applied on dataset of children as presented in Fig. 4.

Results obtained after training local models have been transmitted to central server through 4G ethernet gateway where meta classifier is trained to predict which ML model is outperforming in prediction of ASD. Best model is selected for the training of global model that is transmitted back to the clients so that all clients use same efficient model for autism detection.

#### Analysis and results

Two-dimensional exploratory analysis has been performed on datasets by plotting several graphs to depict different perspectives of the ASD response set. In first dimension, variance between datasets has been analyzed using statistical method ANOVA. ANOVA being a powerful statistical tool compares the mean of datasets and determines that if there is a significant difference between them as summarized in Table 3.

**Table 3:** Analysis of variance summary using ANOVA.

Classes	1	2	3	4	5	Total
Sample (X)	70	70	70	70	70	350
∑X	245	236	2142	245	236	3104
Mean (k)	3.5	3.3714	30.6	3.5	3.3714	8.869
∑X^2^	1155	880	100,132	1155	880	104,202
Std.Dev. (σ)	2.0764	1.1056	22.3888	2.0764	1.1056	14.8222

H_o_ (Null hypothesis) = there is no significant difference between the means of datasets being compared.

H_1_ (alternate hypothesis) = there is a significant difference between the means of datasets being compared.

Results of ANOVA have been listed in Table 4. Total variability of data is calculated by sum of squares (SS). Degree of freedom represent the number of independent observations available to estimate every response. F-statistics and associated p-value are significant results obtained from ANOVA test. F-statistics determines the variability between the groups to the variability within the group. *p* value presents the probability to observe a difference as large as the one observed in response set.

**Table 4:** ANOVA results.

Source	Sum of Squares (SS = Σσ/Σk)	Degree of freedom (df)	Mean square (MS = ∑X^2^/df)
Between-datasets	41,323.47	4	10,330.87
Within-datasets	35,350.49	345	102.4652
Total	76,673.95	349	

The *f*-ratio value is 100.8232. The *p* value is < 0.00001. The result is significant at *p* < 0.05. There is a significant difference between the means being compared. The *p* value is less than the commonly used significance level (0.05), it can be inferred that H_o_ has been rejected and can be concluded that H_1_ has been accepted indicating the significant difference between the means being compared.

**Figure 5:**
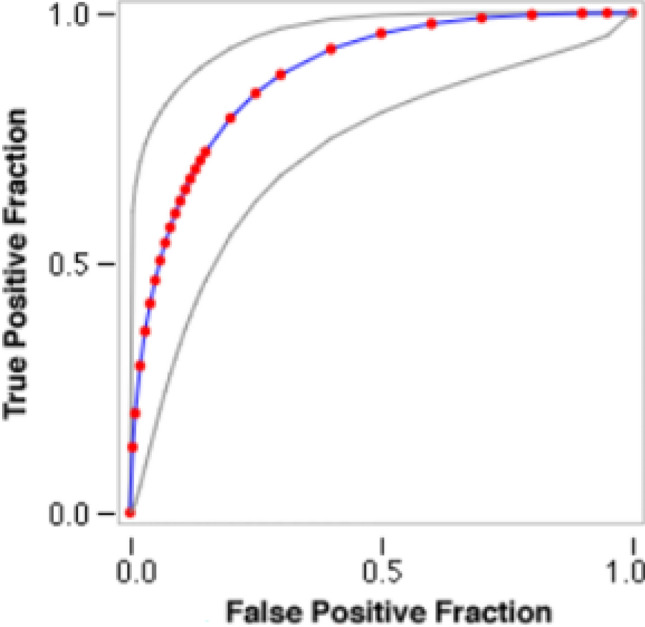
ROC curve.

Second dimension of analysis part focused on visualizing performance of global model trained on central server through meta classifier by drawn receiver operating characteristic (ROC) curve. Data characteristics of ROC curve are presented in Table 5. Figure 5 compares the performance of global model on the basis of sensitivity [TP/(TP + FN)] and specificity [TP/(TP + FN)].

**Table 5:** Data characteristics (ROC curve).

Number of actual negative cases = 31 Number of actual positive cases = 26						
Response data						
Category	1	2		3	4	5
Actual Negative cases	11	12	5	1	2	
Actual positive cases	1	2	4	9	10	
Observed operating points						
FPF	0.0000	0.645	0.968	0.2581	0.6452	1.0000
TPF	0.0000	0.3846	0.7308	0.8846	0.9615	1.0000
Initial values of parameters			Final values of parameters			
A = 1.5647			Procedure converges after 6 iterations			
B = 0.9591			A = 1.7403			
Z(K): − 0.3718 0.6490 1.3003 1.5182			B = 1.1114			
LOGL = − 83.3485			Z(K): − 0.3322 0.5665 1.1380 1.7785 LOGL = − 77.6311			
Variance–covariance matrix			Correlation matrix			
A 0.2089 0.1097 0.0440 0.0520 0.0367 − 0.0012			A 1.0000 0.6688 0.4215 0.4991 0.3029 − 0.0070			
B 0.1097 0.1288 0.0182 0.0048 − 0.0285 − 0.0873			B 0.6688 1.0000 0.2225 0.0592 − 0.3004 − 0.6424			
Z(1) 0.0440 0.0182 0.0521 0.0282 0.0200 0.0110			Z(1) 0.4215 0.2225 1.0000 0.5413 0.3309 0.1272			
Z(2) 0.0520 0.0048 0.0282 0.0519 0.0425 0.0378			Z(2) 0.4991 0.0592 0.5413 1.0000 0.7050 0.4388			
Z(3) 0.0367 − 0.0285 0.0200 0.0425 0.0701 0.0766			Z(3) 0.3029 − 0.3004 0.3309 0.7050 1.0000 0.7642			
Z(4) − 0.0012 − 0.0873 0.0110 0.0378 0.0766 0.1432			Z(4) − 0.0070 − 0.6424 0.1272 0.4388 0.7642 1.0000			
Summary of ROC curve						
Area = 0.8778 Std. dev. (area) = 0.0461

#### Validation

In response set, data points have been gathered into one of the following four classes to validate ASD diagnosis. Class1: true positive (TP) indicates that the person has autism, and we have correctly recorded autism positivity. Class 2: true negative (TN) means that a person does not has autism and wrongly recorded as negative in response dataset. Class 3: false positive (FP) depicts that response dataset incorrectly recorded that a person had ASD who does not have it. Class 4: false negative (FN) indicates that it was predicted mistakenly that the person does not have ASD, but they have ASD. The confusion matrix of ASD that facilitated in the validation process is given below in Table 6.

**Table 6:** ASD prediction matrix.

Detection	Person with ASD	Person without ASD
ASD detected	TP	FP
ASD not detected	FN	TN

Precision, recall and F1 score are the measures used to validate performance of LR and SVM classifiers. Precision demonstrates the cases that detected autism and we predicted them correctly. Whereas recall indicates the number of autism cases identified correctly are relevant out of total instances that had autism. Proposed model has been validated using dataset B, D given in Table1.

**Figure 6:**
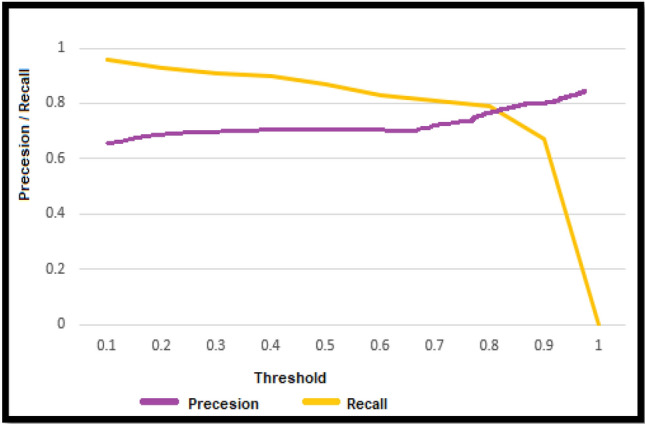
Precision/Recall curve of SVM.

**Figure 7:**
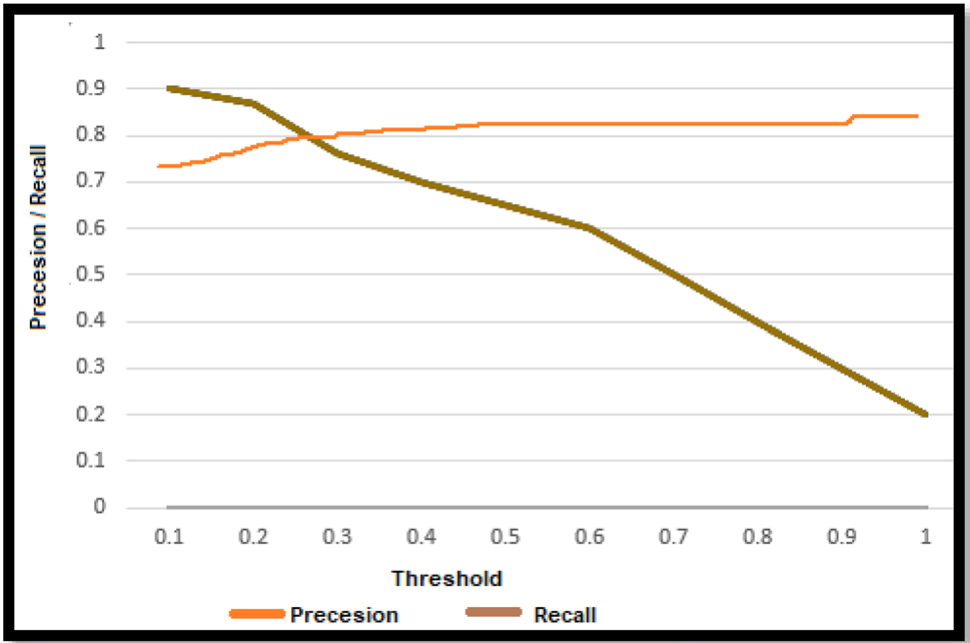
Precision/Recall curve of LR.

F1 score greater than 0.5 or above is considered Good. It can be observed from Table 7 that SVM is performing more accurately than LR although LR is also giving comparable results. Hence, it can be inferred from results that SVM and LR can detect autism more accurately in comparison of other ML models using features dataset and they can be used for early diagnosis of autism. Figures 6 and 7 present precision and recall curve of SVM and LR respectively. Precision and recall are the measures used to evaluate model’s performance. Precision demonstrates the cases that detected autism and we predicted them correctly. Whereas recall indicates how many autism cases model has identified correctly as relevant out of total instances that had autism.

**Table 7:** Performance of ML models in ASD detection.

Matrices	Children dataset		Adult dataset	
	LR	SVM	SVM	LR
Accuracy	98%	99%	81%	78%
Precision	0.92	0.97	0.73	0.81
Recall	0.44	0.58	0.56	0.51
Confusion matrix	$$\left[\begin{array}{cc}24& 2\\ 2& 31\end{array}\right]$$	$$\left[\begin{array}{cc}34& 1\\ 5& 25\end{array}\right]$$	$$\left[\begin{array}{cc}45& 17\\ 14& 35\end{array}\right]$$	$$\left[\begin{array}{cc}30& 7\\ 13& 29\end{array}\right]$$
F1 score	0.60	0.73	0.63	0.62

After performing detailed analysis, it has been observed that SVM and LR models can be best fit for diagnoses of autism disorder in people of various age groups ranging from children to adults. We have obtained 99% accuracy in prediction of ASD.

The performance of proposed model has also been compared with other models already proposed in the literature. We found three most relevant studies that have proposed models for ASD detection.

### Ethical statement

Hereby, I Muhammad Shoaib Farooq consciously assure that for the manuscript “Detection of Autism Spectrum Disorder (ASD) in children and adults using Machine Learning” the following is fulfilled: (1) This material is the authors' own original work, which has not been previously published elsewhere. (2) The paper is not currently being considered for publication elsewhere. (3) The paper reflects the authors' own research and analysis in a truthful and complete manner. (4) The paper properly credits the meaningful contributions of co-authors. (5) The results are appropriately placed in the context of prior and existing research. (6) All sources used are properly disclosed (correct citation). Literally copying of text must be indicated as such by using quotation marks and giving proper reference. (7) All authors have been personally and actively involved in substantial work leading to the paper, and will take public responsibility for its content. The violation of the Ethical Statement rules may result in severe consequences. I agree with the above statements and declare that this submission follows the policies as outlined in the Guide for Authors and in the Ethical Statement.

## Discussion

**Figure 8:**
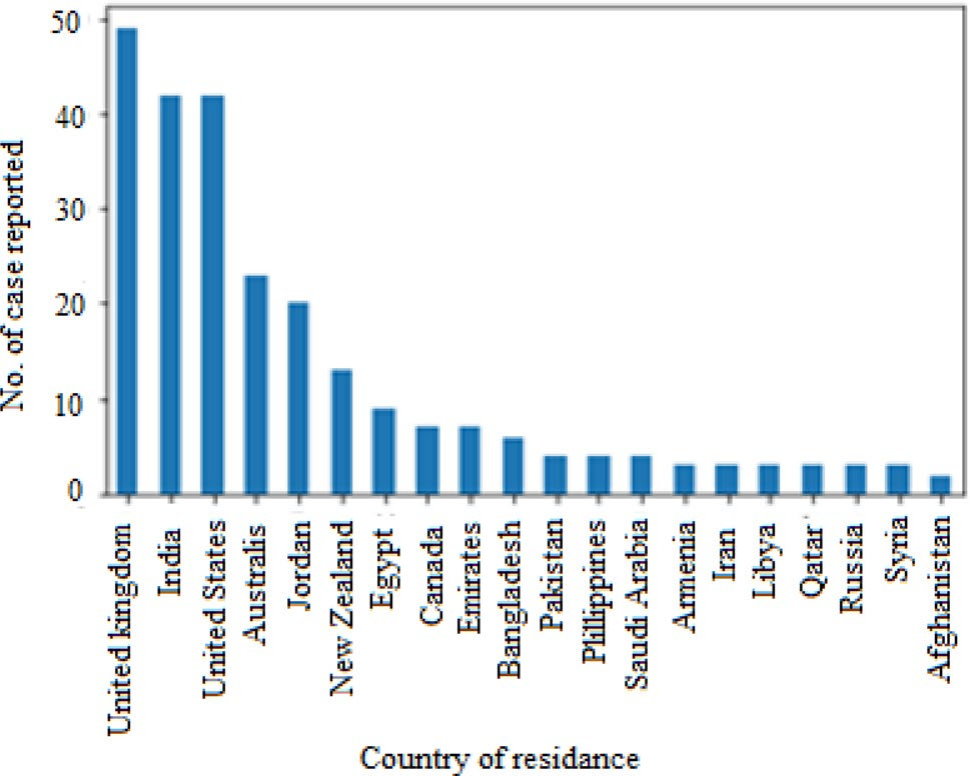
ASD detection as per country-of-residence.

**Figure 9:**
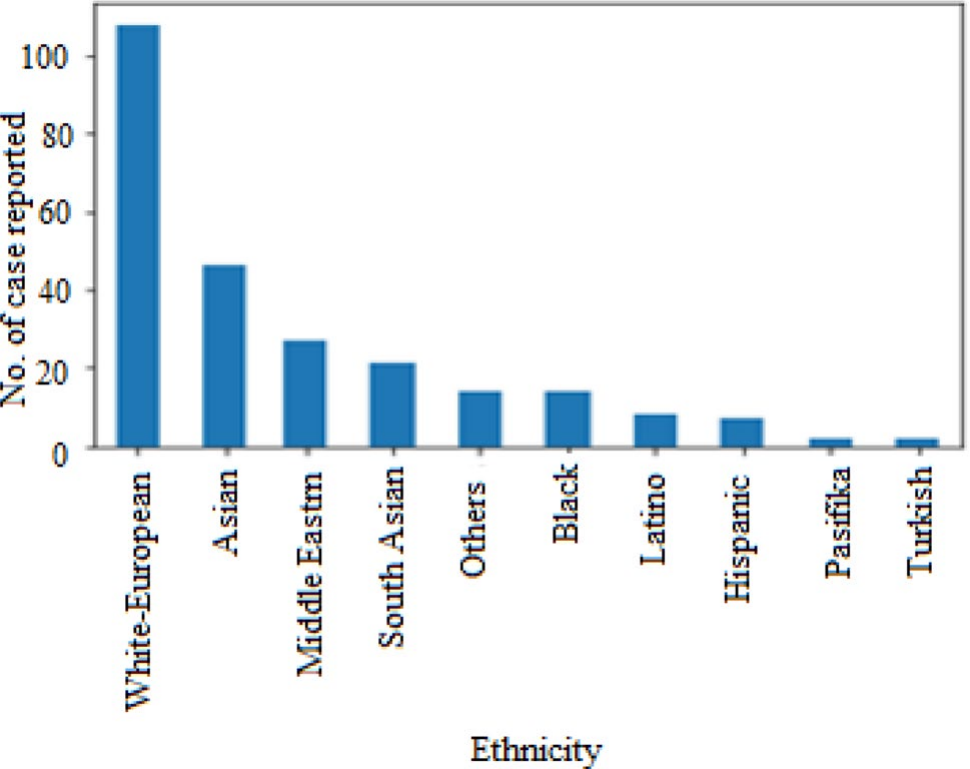
ASD detection as per ethnicity.

Table 2 indicates the response set gathered by analysing multiple features extracted during pre-processing of datasets. Figures 8 and 9 have been drawn based upon response R1 that showed the region to which most of ASD patients belong and their ethnicity. It can be observed from the chart that United Kingdom (UK) is the most affected region. Similarly, graph in Fig. 9 presents that mostly White-Europeans have ASD.

**Figure 10:**
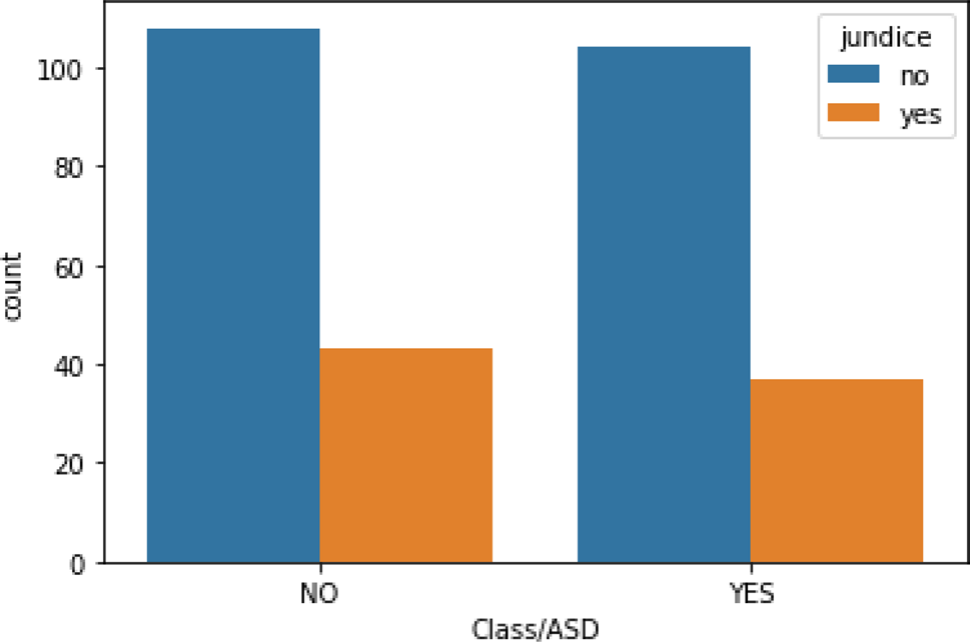
ASD detection based on jaundice.

People infected with jaundice (response R8) are considered as on high risk of ASD. So, it is worthwhile to know that whether a person is born with or without jaundice. There is a high probability that they will screen positive for ASD if born with jaundice as shown in Fig. 10.

**Figure 11:**
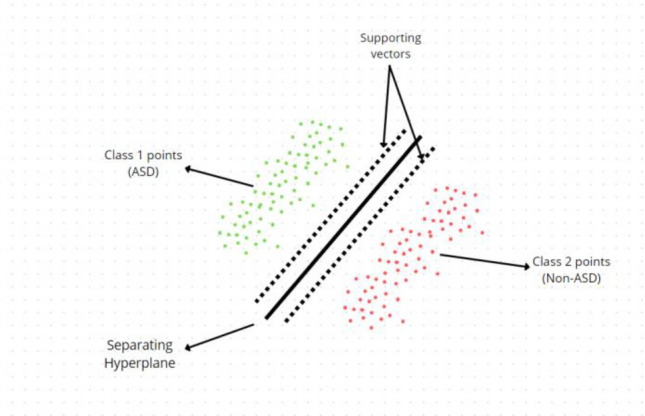
SVM mechanism of ASD Classification.

Application of ML in autism detection has significance due to its reliability, accuracy and quickness^1^. In the proposed model, datasets have been processed to train LR and SVM classifiers locally. Results of these classifiers are transmitted to central server where meta classifier is trained to generate global model for autism detection. The reason for selecting LR is to find a model that most accurately describes the relationship among binary response set and independent variables set^5^. SVMs has been applied in this study as datasets had multiple dimensions and are not linearly separable. SVM use hyperplane that separates ASD dataset into two classes namely ASD effected and Non-ASD to predict target and handle overfitting as well. SVM has separating hyper plane boundary to separate both classes^7^ as presented in Fig. 11.

### Comparison with other studies

We have compared their work with our proposed model and summarized the strengths and limitations of existing model in relation to our proposed models in Table 8. It has been noted that our proposed model is offering comparable accuracy and effectively applicable to diagnose ASD in patients belonging to different age groups ranging from children to adults.

**Table 8:** A comparison of our proposed models with previous work.

Refs	Dataset	Models	Accuracy	Limitations	Strengths
^1^	*Children* 1054 instances along with 18 attributes	LR, SVM, KNN, RF	LR : 93.15% NB:94.79% SVM:93.84% KNN:90.52% RFC: 81.52%	The dataset was compiled from primarily autism-based collections, as a result of which there was quite a significant imbalance, in favor of the ASD class	Used forward feature selection Trained and tested five ML models
^5^	2009 features records (*toddlers, children, adults*)	SVM, Glmboost and adaboost classification	85.10%, 97%, 98%	Constrained sample size/data set	Metrics based on brain activity used for prediction of ASD
^7^	*Children* have 292 instances	Naïve Bayes, SVM, LR, RF, CNN, NN	97.53%, 9 6.30%, 96.88%	Does not predict the severity of ASD. Conditions used for identification of ASD which might not always necessarily translate to a case of ASD	Makes use of six different ML based classification methods and obtained high accuracy
Proposed models	*Children* Records: > 200 Attributes: 22 *Adults* Records: > 700 Attributes: 21	SVM, LR	*Children* SVM: 99% LR: 98% *Adults* SVM: 81% LR: 78%	Size of children dataset is very small	Comparable accuracy has been obtained Two different datasets of children and adults have been processed State of the art FL technique has been applied for ASD prediction

### Limitations of proposed model

FL is a ML technique that allows models to be trained on decentralized data sources without transferring the data to a central server. Proposed FL based model for ASD detection offers several advantages of data security and data privacy but it has some limitations too as listed below:

*Limited model complexity* In proposed architecture, FL models are trained on multiple devices with limited processing power and storage. This limitation can make it difficult to use the proposed model for more complex tasks that require deep neural networks or other advanced machine learning models.

*Data heterogeneity* The proposed model is designed to work with data that is distributed across different devices and locations. However, this can lead to data heterogeneity, where different devices have different types of data, making it challenging to develop models that perform well across all devices.

*Communication overhead* In the proposed architecture, models are trained on local devices, and the updated models need to be sent back to a central server for aggregation. This process can create significant communication overhead, especially when dealing with a large number of devices or when the models are updated frequently.

*Lack of transparency* The proposed model for ASD detection, makes it challenging to understand how models are trained or how they make predictions. This lack of transparency can make it difficult to identify and correct biases or errors in the models.

## Conclusion

The assessment of ASD has been associated with multiple disorders recognized as features including, behavioural, emotional, structural and mental disorders that make it difficult to predict due to non-availability of medical tests for all features needed to detect ASD in a person. Practitioners diagnose ASD in patients by using psychological assessments and response observation. Detection process is time-consuming and complex as symptoms are not obvious. Presently, there is no screening method that has been optimized and thoroughly developed to specifically detect the ASD, nor is there a screening test that can accurately diagnose ASD. ML is the most recent development that can facilitate in predicting autism more accurately saving lots of time. ML can be helpful in early diagnosis of ASD in patients of all ages including children and adults. In this work, we have applied two different ML models (SVM, LR) on the dataset containing features of children and adults. It was observed that SVM showed 81% accuracy in detecting ASD in adults and LR gave 98% accuracy in determining ASD in children. In future, different transfer-learning models i.e. MobileNet, ResNet can also be used in ASD detection using images dataset of autistic children for early detection of ASD with improved accuracy. Moreover, severity of disorder can also be measured through deep learning methods in future.

## Data Availability

Autism image dataset for children : Cihan063, https://www.kaggle.com/datasets/cihan063/autism-image-data, Accessed on: 05 June 2022. Autism Screening on Adults : and rewmvd, https://www.kaggle.com/datasets/andrewmvd/autism-screening-on-adults, Accessed on: 05 June 2022.
